# Six-Month Changes in BMI, Menstrual-Cycle Characteristics, and Ferriman-Gallwey Scores Associated With a Multi-ingredient Nutritional Supplement Blend in Women With PCOS: A Prospective Observational Study

**DOI:** 10.7759/cureus.117455

**Published:** 2026-10-04

**Authors:** Aya Abdelrazzak, Firas K Salman, Ahmad Hammoud, Karl Jallad

**Affiliations:** 1 Department of Emergency Medicine, University Medical Center Rizk Hospital, Beirut, LBN; 2 Department of Internal Medicine, University Medical Center Rizk Hospital, Beirut, LBN; 3 Department of General Surgery, University Medical Center Rizk Hospital, Beirut, LBN; 4 Department of Obstetrics and Gynecology, University Medical Center Rizk Hospital, Beirut, LBN

**Keywords:** bmi, folic acid, hirsutism, inositol, menstrual regularity, n-acetylcysteine, pcos, saw palmetto, α-lipoic acid

## Abstract

Introduction

Multi-ingredient nutritional supplements combining insulin-sensitizing and anti-androgenic compounds are increasingly used in polycystic ovary syndrome (PCOS), yet evidence on their combined clinical effects remains limited. This study aimed to evaluate whether the use of a multi-ingredient nutritional supplement blend containing myo-inositol, α-lipoic acid, N-acetylcysteine, saw palmetto, and folic acid was associated with six-month changes in body mass index (BMI), menstrual-cycle characteristics, and Ferriman-Gallwey hirsutism scores in women with PCOS.

Methods

Thirty-five Lebanese women with PCOS aged 18 to 40 and not receiving metformin or hormonal therapy were enrolled. BMI, menstrual cycle frequency, and Ferriman-Gallwey hirsutism scores were assessed at baseline. Hirsutism was reassessed at two and six months, while BMI and cycle regularity were reassessed at six months. Paired analyses and a linear mixed-effects model for repeated Ferriman-Gallwey scores were applied as appropriate, with significance set at p < 0.05. Three participants were lost to follow-up.

Results

At six months (N=32), mean BMI decreased significantly from 26.5 ± 2.8 to 25.7 ± 2.5 kg/m² among participants with complete paired BMI data (mean change = -0.88 ± 1.56 kg/m²; p = 0.003). Ferriman-Gallwey scores declined from 15.3 ± 3.2 at baseline to 15.1 ± 3.0 at 2 months and 14.4 ± 2.6 at 6 months. In a linear mixed-effects model, the baseline-to-six-month reduction remained statistically significant (mean difference: -1.08; 95% CI: -1.38 to -0.77; Holm-adjusted p < 0.001), while the smaller baseline-to-two-month change did not remain statistically significant after adjustment for multiple comparisons. Menstrual cycle regularity showed a non-significant improving trend (regular cycles: 8.6% to 18.8%; p=0.250).

Conclusions

Six months of using the supplement blend was associated with a modest reduction in BMI and a statistically significant but small baseline-to-six-month reduction in Ferriman-Gallwey hirsutism scores, with no statistically significant improvement in menstrual-cycle regularity. These observational findings should be interpreted as preliminary and support further randomized controlled trials with standardized lifestyle monitoring, objective metabolic and hormonal outcomes, and systematic safety assessment.

## Introduction

Polycystic ovary syndrome (PCOS) is among the most common endocrine and metabolic disorders affecting women of reproductive age. PCOS is diagnosed by the presence of at least two of the Rotterdam criteria: oligo-amenorrhea, clinical or biochemical hyperandrogenism, and polycystic ovarian morphology on ultrasound, in the absence of other endocrinological conditions [1]. The estimated prevalence of PCOS varies according to the diagnostic criteria used, but global prevalence has been estimated at 9.2%, rising to 11.5% when the Rotterdam criteria are applied [2]. In the Middle East, pooled prevalence has been estimated at 8.9% based on NIH criteria and 11.9% in Persian and Levant regions based on Rotterdam criteria [3]. In a Lebanese study of 160 women presenting with hirsutism, PCOS was identified as the underlying cause in 72.6% of participants [4]. Among Lebanese women with PCOS, phenotype A, characterized by hyperandrogenism, ovulatory dysfunction, and polycystic ovarian morphology, has been reported as the most common phenotype [5].

The pathophysiology of PCOS is multifactorial and involves dysregulation of the hypothalamic-pituitary-ovarian axis, ovarian androgen excess, and metabolic dysfunction [6,7]. Increased gonadotropin-releasing hormone (GnRH) pulsatility favors luteinizing hormone secretion, which contributes to ovarian androgen production and ovulatory dysfunction. These endocrine changes contribute to clinical manifestations such as oligomenorrhea, acne, androgenic alopecia, oily skin, and hirsutism [6,7].

Hyperandrogenism and insulin resistance are closely interrelated in PCOS. Excess androgens may contribute to central adiposity, while hyperinsulinemia can exacerbate ovarian androgen production through effects on theca cells and related insulin-like growth factor pathways [6,7]. Impaired glucose tolerance and type 2 diabetes are more prevalent among women with PCOS than among women without PCOS, although these metabolic risks vary by ethnicity and adiposity [8].

Management of PCOS is individualized and targets metabolic dysfunction, ovulatory dysfunction, and hyperandrogenic symptoms. Common approaches include lifestyle modification, insulin-sensitizing therapy, hormonal therapy, ovulation induction when fertility is desired, and anti-androgenic therapy for hyperandrogenic manifestations [6,9].

Among the available therapeutic approaches, nutritional supplementation remains an area of active investigation. The supplement evaluated in this study combines myo-inositol (MYO), alpha-lipoic acid (ALA), N-acetylcysteine (NAC), saw palmetto derived from Serenoa repens, and folic acid. Inositol supplementation has been studied for metabolic, hormonal, and ovulatory outcomes in PCOS [10,11]. ALA has antioxidant and insulin-sensitizing properties and has also been evaluated alone and in combination with MYO in PCOS [12,13]. NAC has been investigated as an adjunctive option for PCOS-related metabolic dysfunction, oxidative stress, and ovulatory outcomes [14,15]. Saw palmetto has been proposed to have anti-androgenic activity through 5-alpha-reductase inhibition, but clinical evidence in PCOS remains limited [16].

Several nutritional supplements and complementary therapies have been proposed for PCOS, including inositols, vitamins, minerals, antioxidants, omega-3 fatty acids, probiotics, and herbal compounds; however, the certainty and clinical applicability of this evidence vary, supporting the need for further investigation before routine integration into clinical practice [9].

Given the insulin-sensitizing, antioxidant, and metabolic effects proposed for myo-inositol, α-lipoic acid, and N-acetylcysteine, and the anti-androgenic activity proposed for saw palmetto through 5-alpha-reductase inhibition, combining these ingredients may plausibly target both BMI-related and hirsutism-related clinical features of PCOS. However, evidence evaluating this combination as a single nutritional supplement blend remains limited. Therefore, this prospective observational study aimed to evaluate whether six months of use of a multi-ingredient nutritional supplement blend containing myo-inositol, α-lipoic acid, N-acetylcysteine, saw palmetto, and folic acid was associated with changes in BMI, menstrual-cycle characteristics, and Ferriman-Gallwey hirsutism scores in women with PCOS. The primary outcomes were changes in BMI and menstrual-cycle characteristics at six months, and the secondary outcome was change in Ferriman-Gallwey hirsutism score at two and six months.

## Materials and methods

A prospective observational study was conducted in Lebanon among women with PCOS recruited from the outpatient Department of Obstetrics and Gynecology at the Lebanese American University Medical Centers - Rizk Hospital (LAUMC-RH) and Saint John’s Hospital (LAUMC-SJH). The study was reported in accordance with the Strengthening the Reporting of Observational Studies in Epidemiology (STROBE) recommendations for observational studies. Eligible patients were sent a text message to inquire whether they could be contacted for research purposes. The study was conducted from November 2024 to May 2025, after IRB approval was obtained. Ethical approval was granted by the Lebanese American University Institutional Review Board (LAU IRB) on October 31, 2024 (IRB #: LAUMCRH.KJ1.31/Oct/2024; IRB Registration # IRB00006954 LAUIRB#1).

This study included women aged 18 to 40 years who were prescribed nutritional supplementation by their gynecologist and agreed to participate. At study enrollment, all participants were adults, and eligibility required current confirmation of PCOS by the treating gynecologist according to the Rotterdam criteria and current international guideline recommendations [1,7]. Reported age at PCOS diagnosis was recorded as historical clinical information and was not used alone to determine study eligibility. Patients were excluded if they were taking insulin sensitizers, such as metformin, oral contraceptive pills (OCPs), or medications that affect endocrine and metabolic profiles such as antipsychotics, selective serotonin reuptake inhibitors (SSRIs), or lithium. Patients with endocrine disorders that may affect the menstrual cycle, including hyperprolactinemia, Cushing disease, thyroid dysfunction, and other clinically suspected alternative causes of menstrual irregularity or hyperandrogenism, were excluded based on the treating gynecologist’s clinical assessment and available medical records. Patients were also excluded if they were pregnant or lactating, or if they were known to have cardiac, renal, pulmonary, or hepatic disorders. Finally, patients who did not agree to participate, could not provide informed consent, or were unable to attend follow-up appointments were excluded.

Overall, 35 patients with PCOS were included in the study. Their gynecologist prescribed two tablets daily of a nutritional supplement, with each tablet containing 500 mg MYO, 60 mg ALA, 300 mg NAC, 35 mg Serenoa repens, and 50 µg folic acid. Participants were instructed to take two tablets daily for six months according to their treating gynecologist’s routine clinical instructions. The product manufacturer, batch or lot number, detailed chemical forms of each ingredient, saw-palmetto extract standardization, and independent quality-testing documentation were not systematically recorded in the study dataset. Lifestyle modification was advised as part of routine clinical care but was not standardized, protocolized, or monitored during the study. Dietary intake, physical activity, other treatments, cosmetic hair-removal practices, and formal adherence monitoring using pill counts, medication diaries, or supplement logs were not systematically recorded.

The primary outcomes were changes in BMI and menstrual cycle characteristics from baseline to six months. The secondary outcome was change in the Ferriman-Gallwey hirsutism score from baseline to two months and six months. Three patients were lost to follow-up at six months.

A standardized survey questionnaire was used to collect demographic characteristics, baseline menstrual-cycle length and regularity according to the International Federation of Gynecology and Obstetrics (FIGO) classification of menstrual-cycle frequency [17], BMI, and hirsutism severity using the Ferriman-Gallwey score [18]. The intervention was started immediately after baseline data collection. Two outpatient follow-up appointments were scheduled: after two months of supplement use to reassess the Ferriman-Gallwey score, and after six months to reassess BMI, cycle regularity, and Ferriman-Gallwey score. Ferriman-Gallwey scoring was performed during outpatient clinic visits by the treating gynecologist using the same scoring method at baseline and follow-up. The same assessor performed the baseline and follow-up hirsutism assessments. Assessor blinding, duplicate scoring, photographic documentation, and formal intra-rater or inter-rater reliability testing were not performed.

The data obtained were analyzed statistically using SPSS software version 21 (IBM Corp., Armonk, NY, USA). Continuous variables were presented as means with standard deviations (SD) or medians with interquartile ranges (IQR), depending on the distribution of the data. Normality was assessed using the Shapiro-Wilk test and visual inspection of Q-Q plots. Categorical variables were summarized as frequencies and percentages. Analyses were performed using available paired data for each outcome. Participants without follow-up measurements for a given outcome were excluded from that paired analysis, and no imputation of missing data was performed. For paired continuous outcomes, the paired t-test was used for normally distributed paired data, while the Wilcoxon signed-rank test was used when normality was violated. Paired binary menstrual-cycle outcomes were analyzed using McNemar-type paired analyses based on participants with both baseline and six-month data. Longitudinal changes in Ferriman-Gallwey hirsutism scores across baseline, two months, and six months were analyzed using a linear mixed-effects model, with time included as a categorical fixed effect and participant included as a random intercept. This model was selected to account for within-participant correlation across repeated measurements and to include all available hirsutism observations despite unequal follow-up numbers. Pairwise contrasts between time points were estimated from the model and reported with 95% confidence intervals and Holm-adjusted p-values to account for multiple comparisons [19]. Statistical significance was set at p < 0.05.

A priori sample-size estimation was performed using G*Power software [20]. The calculation used the t-test family, means: difference between two dependent means matched pairs, with Cohen’s dz = 0.50, α error probability = 0.05, power = 0.80, and a one-tailed test, yielding a minimum required sample size of 27 complete paired observations for the continuous within-participant outcome. To account for potential attrition, 35 participants were enrolled, of whom 32 completed the six-month follow-up. Because the final analyses were reported using two-tailed p-values and the six-month complete-case sample included 32 participants, the study should be considered exploratory and may be underpowered for two-sided hypothesis testing. The sample-size calculation was based on a continuous paired outcome and was not separately powered for binary menstrual-cycle outcomes.

## Results

Baseline characteristics

The 35 patients included in this study had a mean age of 24.0 ± 3.6 years (range: 18-33). The mean reported age at initial PCOS diagnosis was 20.1 ± 4.1 years (range: 10-28); this reflected the historical age at first diagnosis and did not determine study eligibility, as all participants were adults at enrollment and had current PCOS status confirmed by the treating gynecologist. The participants had a mean baseline BMI of 26.7 ± 2.7 kg/m² (range: 21-31), and a mean baseline Ferriman-Gallwey score of 15.3 ± 3.2 (range: 9-22). Most participants were non-smokers (n=30, 85.7%), and 45.7% (n= 16) reported alcohol intake. In terms of PCOS management, most participants (n= 24, 68.6%) had no prior attempts at PCOS treatment, while only a third of the patients (n= 11, 31.4%) had received previous treatment; 14.3% (n=5) had taken oral contraceptive pills, while others used inositol (n=3, 8.6%) or metformin (n=3, 8.6%). At the time of study inclusion, almost all participants (n= 34, 97.1%) were on no form of medical treatment for PCOS. A detailed summary of the baseline characteristics is provided in Table 1.

**Table 1:** Baseline characteristics of the study population Data are presented as mean ± standard deviation (SD) (minimum–maximum) for continuous variables and n (%) for categorical variables. Percentages were calculated using the total study population (N = 35). OCP: oral contraceptive pill

Total	N = 35 (100%)
Characteristic	mean ± SD (min; max)
Age	24 ± 3.6 (18; 33)
Age Diagnosis	20.1 ± 4.1 (10; 28)
BMI at Baseline	26.7 ± 2.7 (21; 31)
Hirsutism Score at Baseline	15.3 ± 3.2 (9; 22)
Characteristic	n (%)
Smoker	
No	30 (85.7)
Yes	5 (14.3)
Alcohol intake	
No	19 (54.3)
Yes	16 (45.7)
Previous treatment	
No	24 (68.6)
Yes	11 (31.4)
Type of previous treatment	
None	24 (68.6)
OCP	5 (14.3)
Inositol	3 (8.6)
Metformin	3 (8.6)
Current medication	
No	34 (97.1)
Yes	1 (2.9)

Changes in BMI

Among participants with complete paired BMI data at baseline and six months (N = 32), mean BMI decreased from 26.5 ± 2.8 kg/m² (range: 21-31 kg/m²) at baseline to 25.7 ± 2.5 kg/m² (range: 20-31 kg/m²) at six-month follow-up. This corresponded to a mean paired change of -0.88 ± 1.56 kg/m² (range: -6 to 2 kg/m²; 95% CI: -1.44 to -0.31; t = -3.172; p = 0.003) (Table 2).

**Table 2:** Paired change in BMI (kg/m²) from baseline to six months among participants with complete paired BMI data (N=32) Data are presented as mean ± standard deviation (SD), minimum–maximum values, and 95% confidence intervals (CI). BMI change was calculated as six-month BMI minus baseline BMI and assessed using a paired t-test. Statistical significance was set at p < 0.05.

Characteristic	mean ± SD	Min - Max	95% CI	Test statistic	P value
BMI at baseline among six-month completers (N=32)	26.5 ± 2.8	21 - 31	25.5-27.5	-	-
BMI at 6 months (N=32)	25.7 ± 2.5	20 - 31	24.7-26.6	-	-
BMI change, 6 months minus baseline	-0.88 ± 1.56	-6 - 2	-1.44- -0.31	t = -3.172	0.003

The distribution of BMI changes among the cohort exhibited interindividual variability (Figure 1). A substantial proportion of participants either experienced no change in BMI (n=14, 43.8%) or had an increase of 1 Kg/m2 and 2 Kg/m2 (n=2, 6.3%; and n=1, 3.1%, respectively). However, a considerable portion of the cohort experienced a decrease in BMI at six months, where 18.8% (n=6) of participants exhibited a reduction of 1 kg/m². Reductions of 2 and 3 kg/m2 were observed in 12.5% (n=4) of participants for each level, and 3.1% (n=1) were found to have a considerable reduction of 6 Kg/m2. The distribution, although delineating a heterogeneous response, aligned with the significant mean paired reduction in BMI at six months by 0.88 kg/m² (p = 0.003).

**Figure 1:**
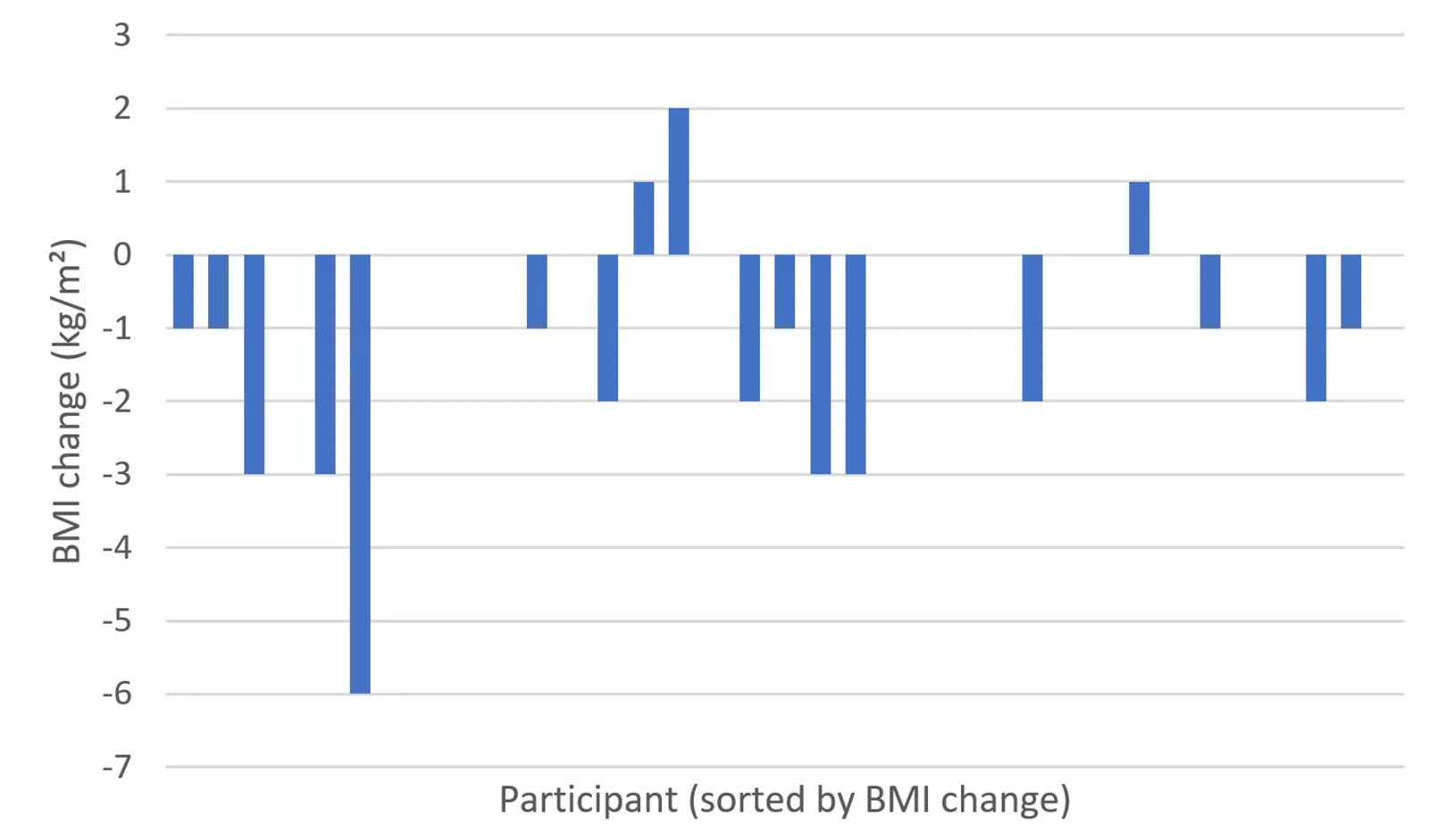
Individual changes in BMI (kg/m²) from baseline to six months of supplementation Each bar represents the change in BMI (kg/m²) for an individual participant after six months of supplementation compared with baseline. Negative values indicate a reduction in BMI, whereas positive values indicate an increase in BMI.

PCOS-related symptoms and cycle frequency

At baseline (N=35), menstrual irregularities were prevalent, with infrequent cycles affecting 68.6% (n=24) of participants, and infrequent cycles with scanty flow being present in 65.7% (n=23). Less common features included amenorrhea (n=6, 17.1%), scanty flow (n=3, 14.3%), and frequent cycles (n=3, 8.6%), whereas only 8.6% (n=3) of patients reported initially having regular menstrual cycles. At the six-month follow-up, there was a non-significant trend toward improved cycle regularity, with less infrequent cycles with scanty flow (n=17, 53.1%, p = 0.250) and infrequent cycles alone (n=19, 59.4%, p = 0.626) being reported by the participants. Regular cycle reporting increased from 8.6% (n=3) at baseline to 18.8% (n=6) at six months (p = 0.250), while other parameters, such as amenorrhea (n=5, 15.6%; p = 1.0), scanty flow (n=5, 15.6%; p = 1.0), and frequent cycles (n=3, 9.4%; p = 1.0), remained closely similar to baseline. McNemar tests confirmed no statistically significant changes in any parameter (Table 3).

**Table 3:** Presence of PCOS-related clinical characteristics at baseline and at six months Data are presented as n (%). Changes between baseline and six-month assessments were evaluated using McNemar tests for paired binary outcomes, with exact two-tailed p-values reported. Statistical significance was set at p < 0.05. PCOS: polycystic ovary syndrome

Total	N= 35 (100%)	N= 32 (100%)	-		-
Characteristics	At baseline n (%)	At 6 months n (%)	Test statistic		P value
Infrequent cycles with scanty flow					
No	12 (34.3)	15 (46.9)	-		-
Yes	23 (65.7)	17 (53.1)	Z= -1.732		0.25
Infrequent cycles					
No	11 (31.4)	13 (40.6)	-		-
Yes	24 (68.6)	19 (59.4)	Z= -1.000		0.625
Amenorrhea					
No	29 (82.9)	27 (84.4)	-		-
Yes	6 (17.1)	5 (15.6)	Z= -1.000		1
Scanty flow					
No	30 (85.7)	27 (84.4)	-		-
Yes	5 (14.3)	5 (15.6)	Z= 0.000		1
Frequent cycles					
No	32 (91.4)	29 (90.6)	-		-
Yes	3 (8.6)	3 (9.4)	Z= 0.000		1
Regular cycles					
No	32 (91.4)	26 (81.3)	-		-
Yes	3 (8.6)	6 (18.8%)	Z= -1.732		0.25

Longitudinal assessment of menstrual parameters revealed stability in most PCOS-related characteristics over the six-month intervention (Table 4, Figure 2). Most participants (n=29, 90.6%) showed no change in infrequent cycles with scanty flow, and 89.7% (n=26) continued having infrequent cycles. Of the participants, 96.9% (n=31) reported no changes related to amenorrhea over 6 months, and all (n=32, 100%) reported no changes in terms of scanty flow and the frequency of their cycles. In addition, lack of regular cycles remained consistent in 90.6% (n=29) of participants, aligning with the observed, non-significant trend towards increased regularity.

**Table 4:** Overall frequency of change in the PCOS-related clinical characteristics after six months Data are presented as n (%). Percentages are based on participants with complete follow-up data (N = 32). PCOS: polycystic ovary syndrome

-	Total N= 32 (100%)
Characteristics	n (%)
Change in Infrequent cycles with scanty flow	
No	29 (90.6)
Yes	3 (9.4)
Change in infrequent cycles	
No	26 (89.7)
Yes	3 (10.3)
Change in amenorrhea	
No	31 (96.9)
Yes	1 (3.1)
Change in scanty flow	
No	32 (100)
Yes	0 (0)
Change in frequent cycles	
No	32 (100)
Yes	0 (0)
Change in regular cycles	
No	29 (90.6)
Yes	3 (9.4)

**Figure 2:**
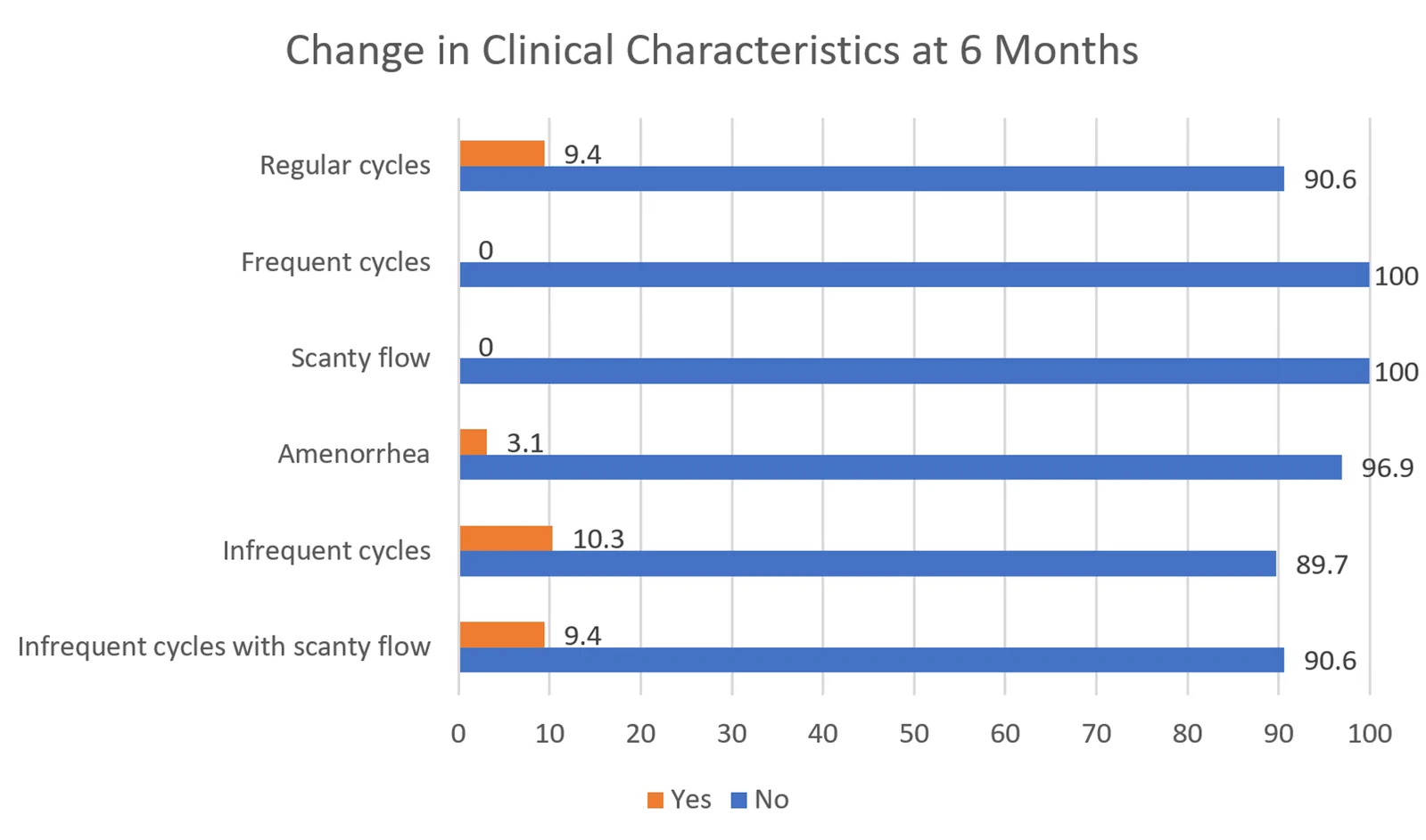
Change in PCOS-related clinical characteristics after six months (Table 4) Data are presented as n (%) for participants with complete follow-up data (N = 32). PCOS: polycystic ovary syndrome

Looking at the type of changes in PCOS-related clinical characteristics after 6 months of follow-up (Table 5), few clinically relevant improvements were seen, as 9.4% (n=3) of participants transitioned to regular cycles (from “No” to “Yes”). Additionally, symptom resolution was seen in 9.4% (n=3) of patients who noted having infrequent cycles with scanty flow, and 10.3% (n=3) of those who noted having infrequent cycles alone (from “Yes” to “No”), though new-onset infrequent cycles developed in one case (n=1, 3.4%). In contrast, all patients remained symptomatic in terms of amenorrhea, scanty flow, and frequent cycles (n=5, 15.6%, n=5, 15.6%, and n=3, 9.4% respectively), with only a single case of amenorrhea having resolved (3.1%). High directional stability was further reflected in the predominance of “No” to “No” transitions (e.g., 81.3% for amenorrhea, 84.4% for scanty flow), and “Yes” to “Yes” states (e.g., 62.1% for infrequent cycles).

**Table 5:** Type of changes in PCOS-related clinical characteristics after six months Data are presented as n (%) and are based on participants with complete follow-up data (N=32). * = Data only available for N=29 for this question. PCOS: polycystic ovary syndrome

	Type of change			
	No to No n (%)	No to Yes n (%)	Yes to Yes n (%)	Yes to No n (%)
Infrequent cycles with scanty flow	12 (37.5)	0 (0)	17 (48.6)	3 (9.4)
Infrequent cycles *	7 (24.1)	1 (3.4)	18 (62.1)	3 (10.3)
Amenorrhea	26 (81.3)	0 (0)	5 (15.6)	1 (3.1)
Scanty flow	27 (84.4)	0 (0)	5 (15.6)	0 (0)
Frequent cycles	29 (90.6)	0 (0)	3 (9.4)	0 (0)
Regular cycles	26 (81.3)	3 (9.4)	3 (9.4)	0 (0)

Ferriman-Gallwey hirsutism scores

Baseline hirsutism severity in the cohort was moderately high, with a mean Ferriman-Gallwey score of 15.3 ± 3.2 (range: 9-22; N = 35). A small descriptive decrease was observed at two months (15.1 ± 3.0; range: 9-22; N = 34), followed by a greater descriptive decrease at six months (14.4 ± 2.6; range: 9-19; N = 32) (Table 6). Formal longitudinal analysis using a linear mixed-effects model is presented in Table 7. This descriptive pattern suggested a greater observed reduction in Ferriman-Gallwey scores at six months than at two months (Figure 3).

**Table 6:** Ferriman-Gallwey score at baseline, at two months, and at six months Data are presented as mean ± standard deviation (SD) (minimum-maximum values).

Characteristics	N	Mean ± SD (min-max)
Hirsutism Score at Baseline	35	15.3 ± 3.2 (9-22)
Hirsutism Score at 2 months	34	15.1 ± 3.0 (9-22)
Hirsutism Score at 6 months	32	14.4 ± 2.6 (9-19)
Change Hirsutism at 2 months	34	-0.29 ± 0.6 (-2-0)
Change Hirsutism at 6 months	32	-1.09 ± 1.1 (-3-0)

**Table 7:** Linear mixed-effects model pairwise contrasts for Ferriman-Gallwey hirsutism scores across follow-up The model included time as a categorical fixed effect and participant as a random intercept. Data are presented as model-estimated mean differences, 95% confidence intervals (CI), z-statistics, and Holm-adjusted p-values.

Comparison	Mean difference	95% CI	Test statistic	Holm-adjusted p-value
Baseline to 2 months	-0.29	-0.58 to 0.01	z = -1.918	0.055
Baseline to 6 months	-1.08	-1.38 to -0.77	z = -7.012	<0.001
2 months to 6 months	-0.79	-1.09 to -0.49	z = -5.137	<0.001

**Figure 3:**
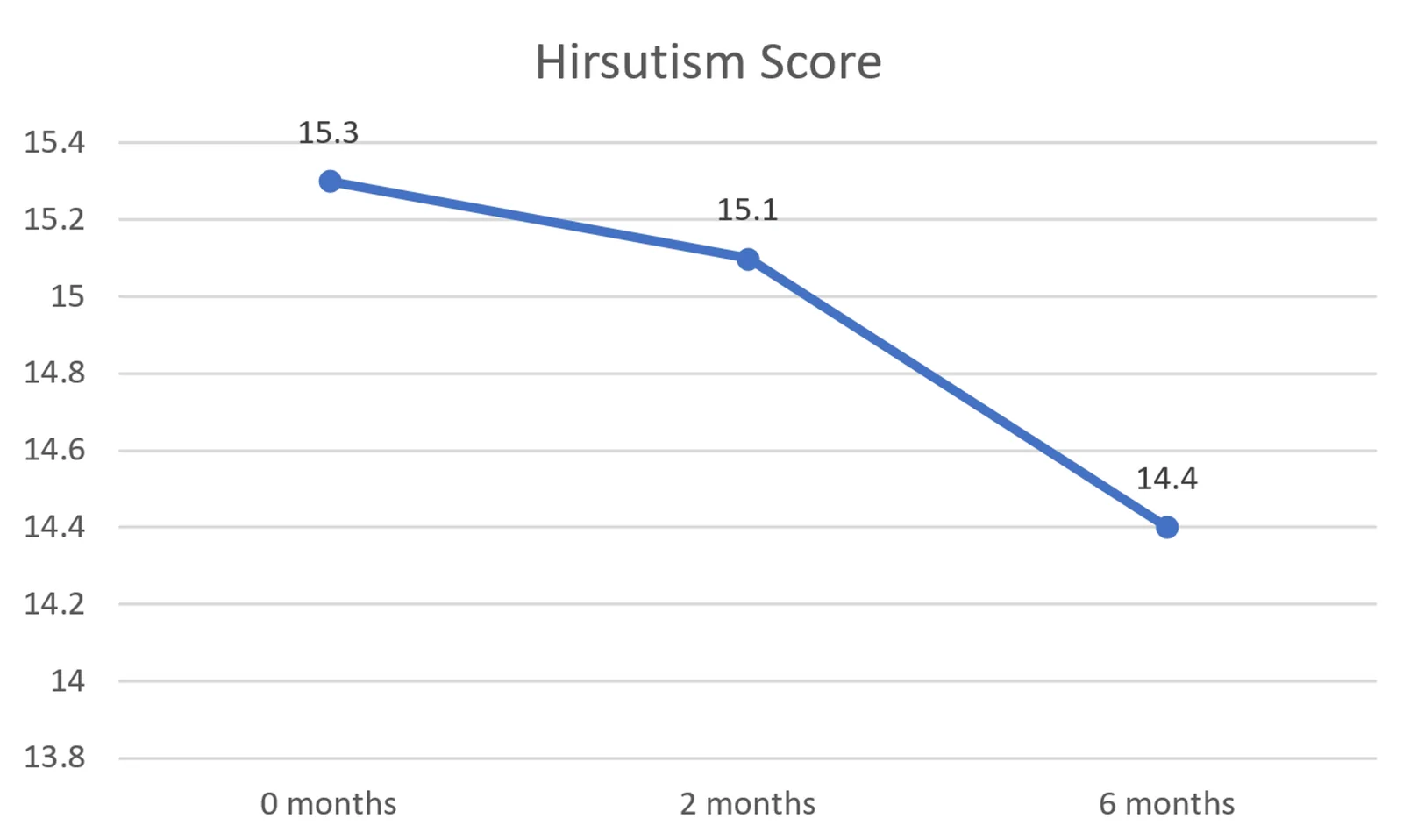
Mean Ferriman-Gallwey score decreasing from 15.3 to 15.1 and to 14.4 at 0, 2, and 6 months, respectively Data are presented as mean Ferriman-Gallwey scores at baseline (N = 35), two months (N = 34), and six months (N = 32).

Figure 4 illustrates the distribution of Ferriman-Gallwey score changes at two and six months. At 2 months, most participants (n=26, 76.5%) exhibited no reduction (0 points on the Ferriman-Gallwey scale), with only 17.6% (n=6) achieving a 1-point decrease, 5.9% (n=2) achieving a 2-point decrease, while none reached a 3-point reduction. By 6 months, the response profile shifted substantially, where a combined 62.5% (n=20) of participants had reductions of 1, 2, or 3 points (n=9, 28.1%; n=7, 21.9%; and n=4, 12.5%, respectively), while no changes were observed in 37.5% (n=12) of the cohort. This visual pattern aligned with the mixed-model findings, in which the baseline-to-six-month reduction remained statistically significant, while the smaller baseline-to-two-month change did not remain statistically significant after adjustment for multiple comparisons.

**Figure 4:**
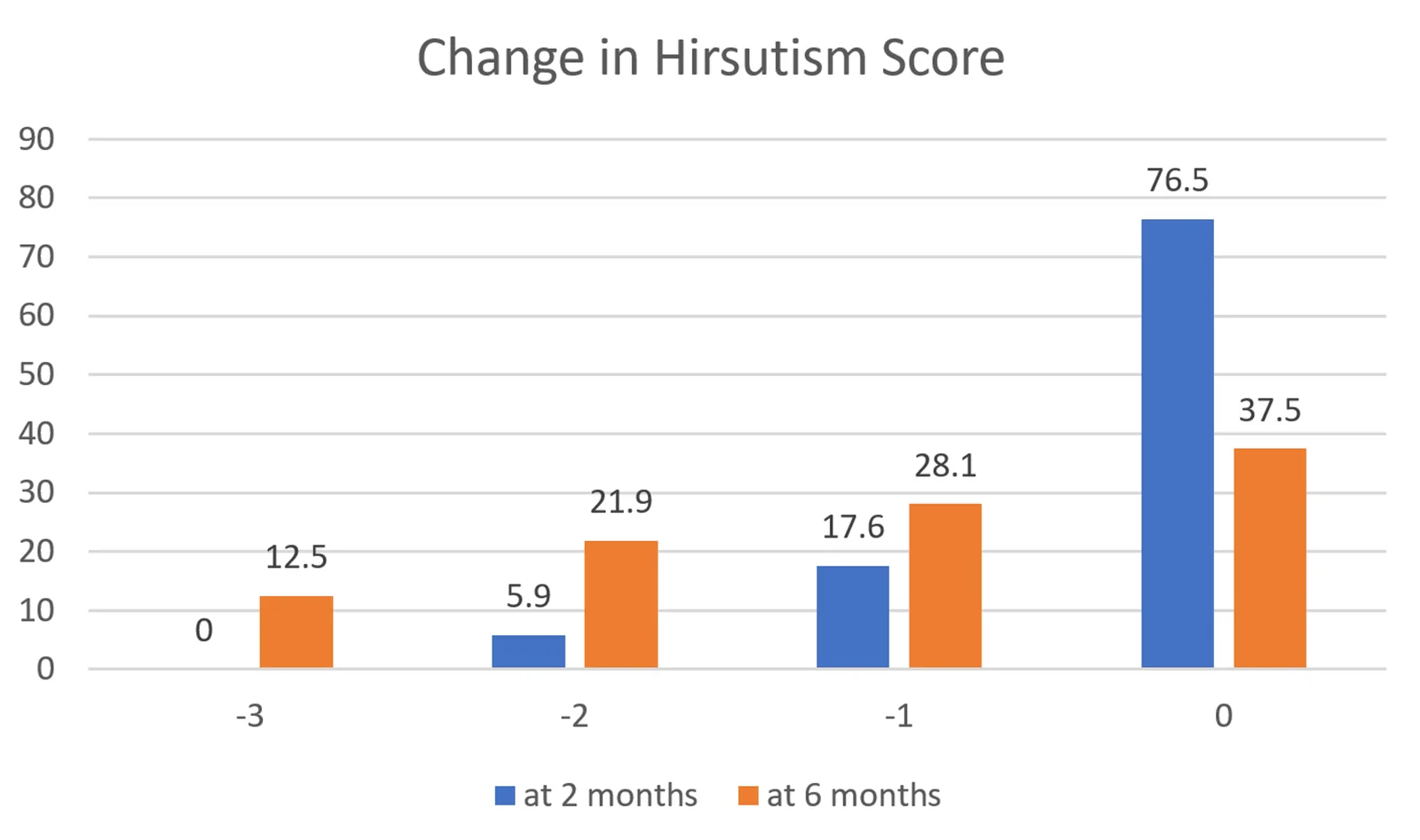
Distribution of changes in Ferriman-Gallwey hirsutism scores from baseline at two and six months of supplementation Data are presented as n (%) of participants according to the magnitude of change in Ferriman–Gallwey score relative to baseline. Percentages were calculated using participants with complete follow-up data at 2 months (N = 34) and 6 months (N = 32).

A linear mixed-effects model was used to evaluate Ferriman-Gallwey hirsutism scores across baseline, two months, and six months while accounting for repeated measurements within participants and unequal numbers of observations across time points. One participant missed the two-month hirsutism assessment, and three participants did not complete the six-month follow-up; therefore, the model used all available hirsutism observations without imputation. In this model, the estimated mean change from baseline to two months was small and did not remain statistically significant after adjustment for multiple comparisons (mean difference: -0.29; 95% CI: -0.58 to 0.01; z = -1.918; Holm-adjusted p = 0.055). The estimated reduction from baseline to six months remained statistically significant (mean difference: -1.08; 95% CI: -1.38 to -0.77; z = -7.012; Holm-adjusted p < 0.001), as did the reduction from two to six months (mean difference: -0.79; 95% CI: -1.09 to -0.49; z = -5.137; Holm-adjusted p < 0.001). No participant with available follow-up data had an increase in the Ferriman-Gallwey score.

## Discussion

This study evaluated six-month changes in BMI, menstrual-cycle characteristics, and Ferriman-Gallwey hirsutism scores associated with the use of a multi-ingredient nutritional supplement blend in women with PCOS. The main findings were a modest reduction in BMI, a statistically significant but small baseline-to-six-month reduction in Ferriman-Gallwey scores, and no statistically significant improvement in menstrual-cycle characteristics. Given the uncontrolled observational design, these findings should be interpreted as within-participant changes over time rather than evidence of efficacy or ingredient-specific treatment effects.

Inositol has been frequently investigated in PCOS, often in comparison with metformin. A 2024 systematic review and meta-analysis found limited and inconsistent evidence suggesting metabolic benefits with MYO or D-chiro-inositol (DCI), with ovulatory improvement observed mainly with DCI [10]. A 40:1 MYO/DCI ratio has been proposed as a potentially favorable formulation for restoring ovulation in PCOS [21]. However, evidence remains mixed. One randomized trial found no added metabolic or hirsutism benefit from MYO combined with metformin compared with metformin alone, although menstrual-cycle improvement was observed [22]. Other studies and meta-analyses suggest that MYO may have effects comparable to metformin for selected metabolic and hormonal outcomes, while combination therapy does not consistently demonstrate superiority over monotherapy [23,24]. Because our study excluded participants taking metformin, MYO provides biological plausibility for further study of BMI- and hirsutism-related outcomes, although the observational design prevents attribution of the observed changes to MYO or to any single ingredient.

Our findings are also consistent with literature evaluating ALA and MYO together. Studies of MYO plus ALA have suggested potential improvements in insulin sensitivity, inflammatory markers, glycemic control, weight-related outcomes, and menstrual-cycle regularity, although findings are not fully consistent across populations and study designs [12,13]. Evidence evaluating ALA alone also suggests potential metabolic benefits, including effects on insulin sensitivity and glycemic parameters, while hormonal benefits appear less consistent [25-27]. These findings provide biological plausibility for further study of this supplement blend. However, the BMI reduction observed in our cohort cannot be attributed to MYO, ALA, NAC, or their combination because lifestyle modification and other potential confounders were not standardized or measured.

NAC has been suggested to have metabolic effects in PCOS, including effects on BMI and insulin-related parameters, with some studies comparing NAC favorably with metformin [28-30]. NAC has also been associated with improvements in ovulatory and hormonal outcomes in women with PCOS, although the magnitude of benefit varies across studies [14,15]. These findings may help explain the limited menstrual-cycle improvement observed in our cohort. However, given the modest sample size, absence of a control group, and use of a multi-ingredient supplement, the contribution of NAC cannot be isolated.

Hirsutism is one of the most distressing hyperandrogenic manifestations of PCOS; however, the observed Ferriman-Gallwey score reduction was small in absolute magnitude. Because no minimally important difference or patient-reported improvement in hirsutism was assessed, the clinical significance of this score reduction remains uncertain. Pharmacologic 5-alpha-reductase inhibition has been studied in PCOS-related hyperandrogenism [31]. Saw palmetto also has proposed anti-androgenic activity through 5-alpha-reductase inhibition, but the evidence base in PCOS remains limited, and dosing is not well standardized [16]. Finasteride and other 5-alpha-reductase inhibitors have been studied for PCOS-related hyperandrogenism, but their use in women of reproductive age requires careful consideration because of potential fetal risk [31,32]. Our study found a statistically significant but small baseline-to-six-month reduction in Ferriman-Gallwey hirsutism score; however, whether this association is related to MYO, saw palmetto, another ingredient, their combination, or unmeasured confounding remains uncertain.

Overall, the observed BMI improvement may have been influenced by MYO, ALA, and NAC, while the reduction in hirsutism may have been influenced by MYO and/or the anti-androgenic properties proposed for Saw palmetto. The lack of statistically significant menstrual-cycle improvement should be interpreted cautiously because the study was not randomized, the cohort was small, and the administered MYO and NAC doses were lower than those used in several prior studies [10,14,15]. Future randomized controlled trials should evaluate this supplement blend against placebo or active comparators, with larger samples, longer follow-up, standardized lifestyle counseling, and outcome measures that can clarify the contribution of each ingredient.

This study has several limitations. First, the observational design and absence of a control group limit causal inference, and the observed changes cannot be attributed to the supplement or to any single ingredient within the blend. Second, the sample size was small, with three participants lost to follow-up, and follow-up was limited to six months, which limits assessment of long-term metabolic, reproductive, hirsutism-related, and safety outcomes. Third, lifestyle modification was advised but not standardized or monitored, and dietary intake, physical activity, cosmetic hair-removal practices, and adherence to supplementation were not systematically recorded, which may have influenced BMI, menstrual cycle, and hirsutism-related outcomes. Fourth, the study relied on clinical outcomes and did not include objective biochemical or cardiometabolic measurements such as waist circumference, blood pressure, fasting glucose, insulin-resistance indices, lipid profile, testosterone, sex hormone-binding globulin, or free-androgen index. Fifth, although Ferriman-Gallwey scoring was performed by the same assessor at each time point, assessor blinding, duplicate scoring, photographic documentation, and formal intra-rater reliability testing were not performed, which may introduce measurement variability. In addition, although all participants were adults at enrollment and had current PCOS status confirmed by the treating gynecologist, the original diagnostic criteria applied at the time of initial PCOS diagnosis, age at menarche, phenotype distribution, biochemical androgen measurements, and detailed laboratory values used to exclude alternative endocrinopathies were not systematically available in the study dataset. This limits the diagnostic granularity of the cohort. Pregnancy and lactation were exclusion criteria at baseline, but longitudinal pregnancy testing, contraception use, contraception counseling, adverse events, discontinuations, laboratory monitoring, and predefined stopping criteria were not systematically recorded during follow-up; therefore, safety conclusions cannot be drawn. Finally, menstrual cycle outcomes were based on participant-reported cycle characteristics rather than objective ovulation measures, which may be subject to recall or reporting bias.

## Conclusions

This prospective observational study found that six months of supplementation with a multi-ingredient nutritional blend containing MYO, ALA, NAC, saw palmetto, and folic acid was associated with a statistically significant but modest reduction in BMI and a statistically significant but small baseline-to-six-month reduction in Ferriman-Gallwey hirsutism scores among Lebanese women with PCOS. Menstrual-cycle regularity showed a non-significant improving trend. Because this study did not include a control group or objective biochemical, metabolic, and androgen measurements, conclusions should be restricted to observed changes in BMI, menstrual-cycle characteristics, and clinically assessed hirsutism scores.

These findings should be interpreted as preliminary because of the small sample size, lack of randomization, absence of a control group, non-standardized lifestyle modification, and inability to isolate the effect of individual ingredients. Larger randomized controlled trials are needed to determine efficacy, assess clinical significance, evaluate optimal dosing and long-term safety, and clarify whether any observed effects are driven by individual components or by synergistic interactions within the supplement blend.
